# A Sled Dog Model for Positive Health Effects of Weight Management and Exercise

**DOI:** 10.3844/ajbbsp.2023.47.54

**Published:** 2023-02-23

**Authors:** Laura Falkenstein, Scott Jerome, Emma Jerome, Kali Striker, Arleigh Reynolds, Lawrence Duffy, Kriya Dunlap

**Affiliations:** 1 Department of Chemistry and Biochemistry, University of Alaska Fairbanks, Fairbanks, USA; 2 Department of Arctic Biology, University of Alaska Fairbanks, Fairbanks, USA; 3 Department of Psychology, Western Washington University, Bellingham, USA; 4 Department of Veterinary Medicine Program, the University of Alaska Fairbanks, Fairbanks, USA

**Keywords:** Metabolic Syndrome, Sled Dogs, Type II Diabetes, Weight Gain, Weight Loss

## Abstract

Obesity is a significant risk factor for metabolic syndrome and type II diabetes. Physical activity and/or dietary modification can reduce the incidence of obesity. Marginal fitness levels limit the efficacy of exercise training, and most humans choose from a wide range of dietary strategies to lose weight. Despite these limitations, exercise in the absence of weight loss may offer protective benefits against the development of metabolic syndrome and type II diabetes. To examine the benefits of exercise training with and without weight loss, we measured changes in metabolic indices in conjunction with moderate body weight gain, exercise training with no intended change in body weight, and exercise training with bodyweight reduction in a healthy canine model. We measured glucose transporter type 4 on peripheral blood mononuclear cells, plasma glucose, hemoglobin A1c, tumor necrosis factor-alpha, interleukin 6, and insulin levels before, during, and after weight gain, exercise, and weight loss. Weight gain increased plasma glucose, while exercise increased glucose transporter by 4% in peripheral blood mononuclear cells. We also observed changes to plasma glucose, glucose transporter 4, and tumor necrosis factor-α which may be indicative of reduced insulin sensitivity with exercise and weight loss, potentially due to the high energy demand coupled with low caloric availability.

## Introduction

Obesity is an accumulation of excess adipose tissue in the body (WHO, 2020). From birth until adolescence, human white adipose tissue grows through hyperplasia. In adulthood, the number of adipocytes remains stable regardless of body fat changes and adipose tissue grows through hypertrophy instead (Choe *et al*., 2016). Adipocytes are designed for dynamic energy storage, allowing free fatty acids to be stored or released as needed in the body for energy. In obesity, adipocytes become over-enlarged, which can cause cell membrane damage (Pietiläinen *et al*., 2011). This damage leads to cell necrosis, triggering the body’s immune response. Necrotic cells attract macrophages which increase pro-inflammatory cytokines, including Tumor Necrosis Factor-alpha (TNF-α) and Interleukin-6 (IL-6), and decrease the production of adiponectin, disrupting the effective regulation of glucose metabolism (Choe *et al*., 2016). Adipose membrane dysfunction can also lead to disruption in glucose transporter type 4 (GLUT4) trafficking, which is required for insulin signaling (Choe *et al*., 2016). Because of these immune responses and disruptions in insulin signaling, obesity often leads to Metabolic Syndrome (MetS) and Type 2 Diabetes (T2D).

Approximately 25% of adults worldwide and 33% of adults in the U.S. are living with MetS and over 10% of American adults and 9% of adults worldwide are living with T2D (DHHS, 2020; IDF, 2006; WHO, 2020). T2D and its complications are one of the leading causes of premature death in the developed world (IDF, 2006). Fewer than half of T2D patients successfully manage their condition (García-Pérez *et al*., 2013). A diagnosis of T2D or MetS often comes with a recommendation of sweeping lifestyle changes that can be difficult to maintain, which may contribute to the inability to regain glycemic control (Brown *et al*., 2022; García-Pérez *et al*., 2013). The most effective changes are the attainable ones. If we can narrow down the effectiveness of these lifestyle adjustments, there is a greater likelihood that a person at risk of these conditions will be able to make the necessary changes. Exercise has many benefits, but diet generally plays a greater role in actual weight reduction. If the deleterious effects of weight gain can be combatted by exercise alone, then it might be easiest for a patient to increase physical activity without specifically worrying about weight loss. The goal is to take steps to improve outcomes for those at risk for or experiencing symptoms of MetS and T2D. By narrowing the effectiveness of these changes and concentrating on which are most effective, we can improve patient outcomes.

Several studies have examined the effects of weight loss induced by equivalent caloric deficits achieved through either calorie restriction, exercise, or a combination of the two (Coker *et al*., 2009; Lefevre *et al*., 2009; Magkos, 2022; Redman *et al*., 2007). These studies show that total weight reduction occurs fairly equally regardless of the method of energy balance. However, while all methods of weight loss provide health benefits and reduce the risk of obesity-related comorbidities, exercise additionally improves cardiovascular and metabolic health (Redman *et al*., 2007) and additional improvements in markers related to insulin signaling (Coker *et al*., 2009; Redman *et al*., 2011). Despite the superior metabolic benefits of exercise-induced weight loss in obese individuals, marginal fitness levels and inadequate compliance to exercise training may limit the practical aspect of exercise training as a mono-therapy for obesity (Coker and Wolfe, 2018). On the other hand, even modest weight loss or exercise training without weight loss may yield measurable health improvements (Coker *et al*., 2006; 2009; Ryan and Yockey, 2017). Smaller, targeted, achievable goals may produce better results simply because of better patient compliance.

To evaluate the influence of interventions such as exercise training and dietary modifications on the risk of metabolic disease, insulin sensitivity is often measured to evaluate their effectiveness. Unfortunately, many of these approaches are somewhat complicated or lack the specificity required for this type of assessment (Hays *et al*., 2006). GLUT4 is typically found in intracellular storage vesicles which are translocated to the cell surface in response to a stimulus. In T2D, lower GLUT4 levels are found in adipose tissue but not skeletal muscle. Therefore, reduced GLUT4 translocation, rather than global GLUT4 level, is considered to be a significant contributor to insulin resistance (Stöckli *et al*., 2011). GLUT4 in skeletal muscle tissue was initially known to respond to insulin as a stimulus but was also found to translocate in response to exercise (Ren *et al*., 1994). Traditionally, GLUT4 activity has been sampled from biopsied skeletal muscle tissue, which is an invasive procedure. Maratou *et al*. (2007) found that GLUT4 translocation in response to insulin can also be measured in the Peripheral Blood Mononuclear Cells (PBMC). We have previously demonstrated that GLUT4 translocation occurs in PBMC in response to exercise similarly, providing convincing evidence for the use of PBMC as a less-invasive proxy tissue for GLUT4 measurement (Schnurr *et al*., 2014; Sticka *et al*., 2018). Earlier studies measured GLUT4 on PBMC using flow cytometry (Maratou *et al*., 2007), but we have established a protocol to conduct these measurements with a commercially available ELISA, providing a simpler and less expensive approach (Schnurr *et al*., 2014; Sticka *et al*., 2018).

Canines have been used as a pre-clinical model for research in diabetes and metabolism for at least 120 years, with the discovery of an anti-diabetogenic factor produced by the pancreas (Mering and Minkowski, 1890). Diabetes is also common and a growing concern in pet dogs as it is in humans (Catchpole *et al*., 2005). Canines provide a more homogenous population than humans, with an easily controlled diet and exercise routines. Sled dogs especially are elite athletes and therefore at low risk of developing T2D and other obesity-related conditions, so observed changes in biomarkers are likely due to moderate changes in weight and exercise rather than from another underlying condition. These animals have significantly higher basal metabolic rates than humans (Hinchcliff *et al*., 1997) providing an efficient model for observing biomarker shifts. Our lab has recently used a sled dog model to investigate GLUT4 translocation with both acute and chronic exercise (Schnurr et al., 2014; 2015) and changes in TNF and adiponectin with weight gain and exercise separately (Collin et al., 2019). The present study aimed to further investigate biomarkers associated with T2D and MetS in conjunction with weight gain, exercise, and weight loss.

## Materials and Methods

### Test Subjects

**Fig. 1:**
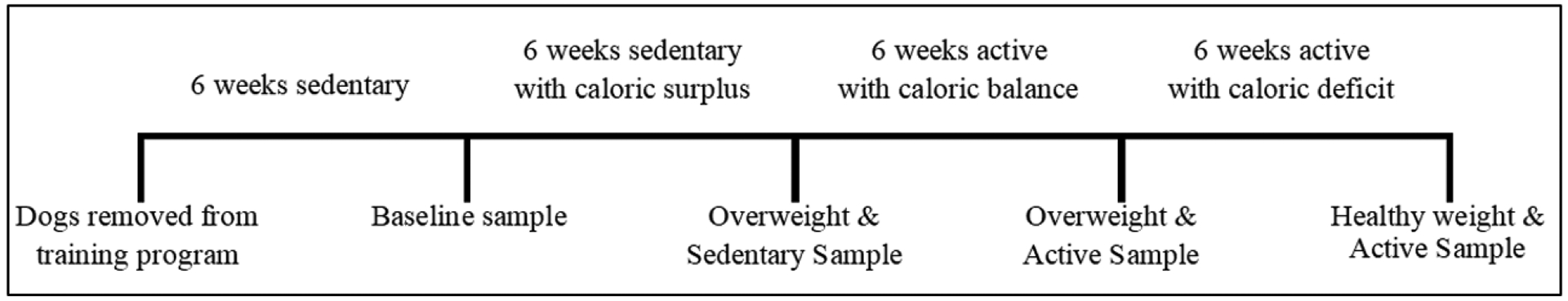
Timeline of diet and exercise intervention and sample collection

**Fig. 2:**
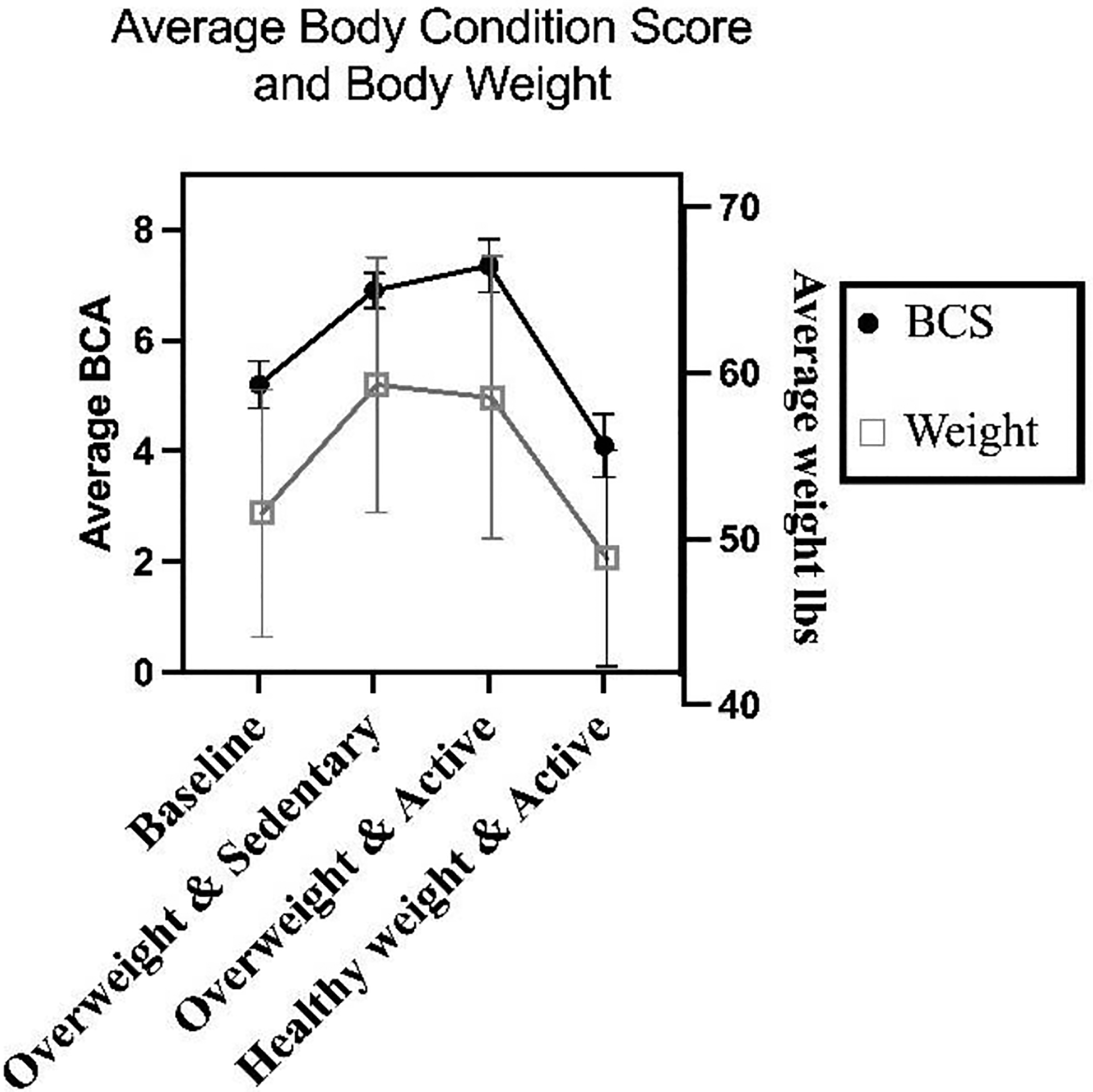
Changes in average BCS and body weight of subjects throughout the study

The subjects examined in this study were privately owned sled dogs housed in Salcha, Alaska (65°N, 147°W). Dogs selected for this study were removed from an athletic training plan for a minimum of six weeks before the study (Fig. 1). Original Body Condition Scores (BCS) ranged from 5–6 upon the baseline blood draw, indicating an ideal to overweight condition (Table 1, Fig. 2) (Laflamme, 1997). Dogs remained sedentary and were fed a caloric surplus for six weeks to allow moderate weight gain bringing the BCS range to 6–7. After six weeks, the dogs began an exercise program, steadily increasing in length and intensity, while fed a caloric balance. All dogs maintained or increased BCS during this phase, bringing the range to BCS of 7–8. After six weeks, feeding was reduced to allow a calorie deficit, while maintaining the exercise plan. After six more weeks, all dogs returned to a weight lower than that at which they began the study, with final BCS ranging from 3–5, indicating a body condition between thin and ideal (Laflamme, 1997).

### Blood Sampling

Blood sampling was completed at baseline, after six weeks of sedentary weight gain, after six weeks of exercise training while overweight and after six weeks of exercise training with weight loss (Fig. 1). Each sample was collected after an overnight fast. Samples were 10 mL of blood drawn from the cephalic vein, 8 mL into a Mononuclear Separation Tube (MST), with the remainder into EDTA vacutainers.

Blood samples collected in MSTs were centrifuged at 3600 rpm for 15 min and plasma was removed, separated, and frozen at 80°C for later analysis. The PBMC were transferred into 15 mL conical vials, re-suspended in 3 mL of RPMI with 5% calf serum, and centrifuged at 1500 rpm for 15 min. The supernatant was discarded and the pellet was re-suspended in 3 mL of RPMI with 5% calf serum for a total of 3 washes. Finally, the PBMC was re-suspended in 2 mL of RPMI with 5% calf serum and immediately analyzed for GLUT4.

Samples collected in EDTA were centrifuged and the plasma was frozen at 80°C for later analysis.

### Analysis

PBMC was standardized for protein content with a BCA assay (thermo fisher scientific) and then analyzed for GLUT4 content with a sandwich ELISA (My BioSource) using the manufacturer’s protocol. IL-6 and TNF-α were also measured by sandwich ELISA (R&D Systems). The sandwich ELISA provided a color complex with absorbance directly proportional to the concentration of the target biomarker. HbA1c was analyzed with a competitive ELISA (My BioSource), which provides an absorbance inversely proportional to the HbA1c concentration. Plasma glucose was measured with a quantitative glucose assay (Abcam), with a measured absorbance directly proportional to the glucose in the sample. Absorbances were measured using a Synergy HT multi-mode microplate reader (Bio Tek). Sample concentrations were determined by interpolations from a standard curve of known concentrations.

### Statistics

Data were analyzed by GraphPad Prism statistical analysis software (version 9.0.1) using one-way ANOVA with Tukey post hoc analysis. All results are expressed as mean ± SD. Differences were considered significant at P≤0.05.

## Results

**Fig. 3:**
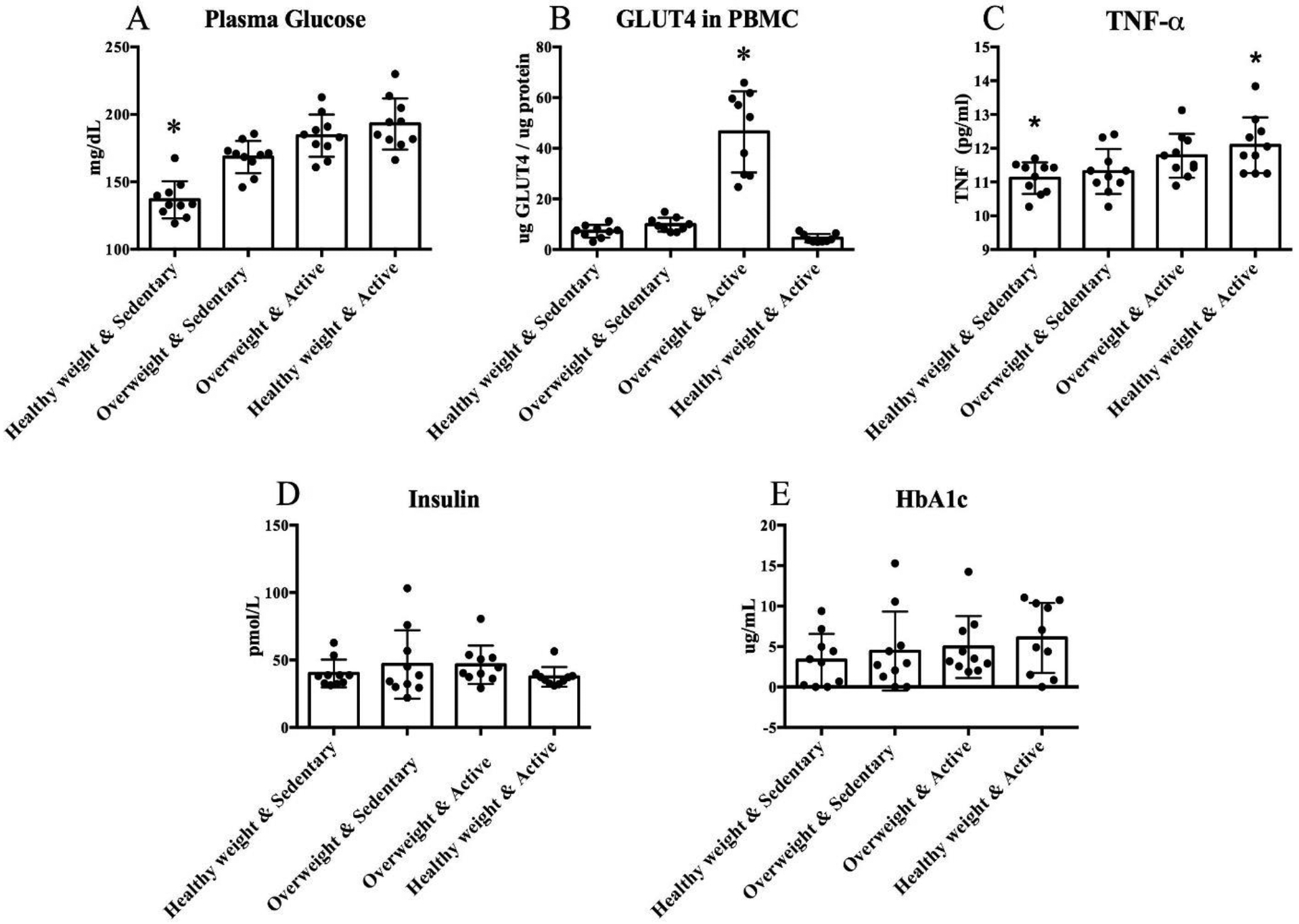
Measured changes in biomarker concentration throughout the study. All data points are shown. Columns show mean ± SD. Significant differences indicated by * (p≤0.05)

Plasma glucose levels rose significantly from the baseline with sedentary weight gain, but there were no significant changes with exercise training with or without weight loss (Fig. 3A). PBMC GLUT4 measurements were significantly higher while active without weight loss, with no significant differences between other samples (Fig. 3B). TNF-α was significantly higher after training with weight loss than at baseline, with no significant differences between other measurements (Fig. 3C). No significant changes were found between samples in measurements of HbA1c or insulin (Fig. 3D–E). All measurements of IL-6 fell below the lowest standard and were unable to be determined.

## Discussion

We observed an increase in plasma glucose levels after sedentary weight gain, which is indicative of a decrease in insulin sensitivity. This is consistent with what is expected in body weight gain. While we might expect insulin signaling to improve with exercise training and therefore plasma glucose to decrease, previous studies have shown that plasma glucose remains relatively unaffected by exercise, but is more responsive to diet (Houmard *et al*., 2004; Shaw *et al*., 2006; Zheng *et al*., 2016). Our data are consistent with these studies. It would follow, then, that we would expect to see a decrease in our sample upon weight loss. While this was not the case in our study, Tvarijonaviciute *et al*. (2012) found similar results in a study of weight loss in obese dogs. When we took our final blood samples, the dogs had returned to an athletic body condition, but were still in a period of active weight loss, on an exercise program while working in a caloric deficit. Animals are known to adapt in periods of starvation by increased gluconeogenesis (Yu *et al*., 2016) and decreased glucose usage to prolong survival (Cahill Jr and Owen, 1968). While the dogs were not in any danger of actual starvation, it is possible their bodies were adapting to the high energy demand and low energy supply.

GLUT4 concentration in PBMC increased as expected when dogs began the exercise program. This reflects what we had found in a previous study of GLUT4 in PBMC of sled dogs (Schnurr *et al*., 2014), providing further evidence that GLUT4 translocation responds not only to insulin, but also to exercise. We expected to see the highest GLUT4 concentrations after weight loss when the dogs were on an exercise program and a healthy body weight, however, GLUT4 levels were significantly lower than they had been while active and overweight. Fasting has been shown to cause a decrease in GLUT4 translocation in human white adipose tissue and rats, possibly due to a decrease in mRNA production (Alves-Wagner *et al*., 2009; Kahn, 1992). While the dogs were not fasting, the high energy demand required by the exercise program coupled with the caloric deficit may have triggered a fasting-like metabolic state.

We expected to see a rise in TNF-α after sedentary weight gain and a decrease while active and at a healthy weight (Choe *et al*., 2016). However, we saw no significant change in weight gain. This is in contrast to our previous findings examining changes in biomarkers upon weight gain (Collin *et al*., 2019). Like Collin *et al*. (2019) we intended to begin this study with dogs at a BCS of 3–4, representing an athletic body condition. In the previous study, there was difficulty getting some dogs to eat enough excess food to gain weight, so this time dogs were specifically selected as “good eaters”. This may have played a role in sedentary dogs beginning the study at a higher BCS than intended, potentially downplaying the effects of weight gain on TNF-α. Additionally, the dogs tested by Collin *et al*. (2019) maintained a higher weight for a longer period before being sampled, waiting for two of the dogs to gain weight. The subjects in the present study were still in a phase of metabolic transition when sampled, possibly accounting for the difference in observations.

We also saw a rise in TNF-α while active at a healthy weight. At the time of the final blood samples, the dogs were active in a caloric deficit, which could put extra stress on the body, increasing the expression of TNF-α. Like the observations of increasing plasma glucose, an increase in inflammatory biomarkers including TNF-α may be a response of the body functioning in a caloric deficit. Fernández-Real and Ricart (1999) describe insulin resistance and inflammation in humans as a protective adaptation in times of famine. Strenuous exercise has also been acknowledged to upregulate pro-inflammatory cytokines (Pedersen and Nieman, 1998). The increase in pro-inflammatory biomarkers we see here may very well be representative of strenuous exercise and a caloric deficit, rather than changes in body fat.

Moderate weight gain produced effects indicative of decreased insulin sensitivity to some, but not all, biomarkers measured. The exercise provided a dramatic shift in GLUT4 translocation, but no significant changes to other biomarkers, while exercise training with weight loss appeared to reverse the effect. Collecting our final samples while the dogs were still in a caloric deficit likely skewed these results. Another six-week phase with continued exercise but a diet fed to maintain weight may provide better insight into the effects of activity at a healthy weight. These results also open the door to a question regarding the potentially deleterious effects of rapid weight loss. The three biomarkers observed here point to potential inflammation and reduced insulin signaling during a period of high activity with reduced caloric intake. Future studies should explore other inflammatory biomarkers and potential long-term effects of rapid weight loss.

## Conclusion

Weight gain induced a rise in plasma glucose levels while exercise increased the translocation of GLUT4 to the PBMC. Continued exercise training paired with a caloric deficit showed detrimental effects on plasma glucose levels, GLUT4 translocation, and TNF-α. Further studies should examine the effects of exercise coupled with caloric restriction on inflammation and insulin signaling with a longer timeline. The beneficial effects of exercise, paired with ideal body weight may have been masked by the caloric deficit in the diet. A better assessment of exercise at a healthy weight would be to have the animal maintained at a healthy weight for a minimum of 6 weeks.

## Figures and Tables

**Table 1:** BCS and body weights of subjects at each sample collection

Dog	Baseline		Overweight and sedentary		Overweight and active		Healthy weight and active	
	BCS	Weight (lbs)	BCS	Weight (lbs)	BCS	Weight (lbs)	BCS	Weight (lbs)
1	5	58.6	7	65.1	7.0	63.0	3	52.3
2	5	53.2	7	60.2	7.0	57.9	4	46.8
3	6	55.9	7	64.3	8.0	65.2	4	52.1
4	5	60.5	7	70.2	8.0	69.6	4	56.2
5	5	58.1	7	62.4	8.0	65.4	5	57.4
6	5	40.8	6	49.9	7.0	45.0	4	40.5
7	5	43.2	7	51.5	7.0	50.4	4	41.1
8	5	42.8	7	51.6	7.0	53.1	4	42.7
9	5	55.9	7	67.0	7.5	66.1	5	55.2
10	6	46.6	7	50.6	7.0	49.5	4	44.1
